# Students’ response to a Massage & Meditation medical school course elective

**DOI:** 10.15694/mep.2020.000028.1

**Published:** 2020-02-13

**Authors:** Maxwell Binstock, Leora Morinis, Shelley Adler, Wolf E. Mehling

**Affiliations:** 1 University of California San Francisco and Berkeley; 2 University of California San Francisco

**Keywords:** Curriculum Development, Massage, Mindfulness, Meditation, Medical Education, Burnout, Body Awareness, Medical Students

## Abstract

This article was migrated. The article was marked as recommended.

**Objectives:** Due to high incidence of medical student and physician burnout, medical education needs to include skills for life-work balance. Patients complain that clinicians depend on technology during clinic visits, and use less touch. To address this educational need, we designed an 18-hour curriculum that combines massage (to reduce anxiety and teach skillful touch) and meditation (for burnout prevention). We explored whether learning basics of massage and meditation could give medical students tools for self-care and skillful touch.

**Methods:** The curriculum was implemented as an elective at the Medical School since 14 years. We collected 181 anonymous student evaluations and conducted pre-post surveys to evaluate the curriculum. We assess mindful bodily awareness (by Multidimensional Assessment of Interoceptive Awareness questionnaire) and conducted thematic analysis of students’ comments.

**Results:** Students appeared highly satisfied with the class (4.94 [Range 1-5]) and reported confidence in being able to apply massage and meditation in their personal and professional life. They commented on the importance of skillful touch and gained more confidence in using touch in clinical care. The pre-post survey showed improvements in interoceptive bodily awareness. Students felt that they developed new skills for self-care and stress management, experienced a sense of community among peers, and stated that the class provided necessary teaching complementary to the mandatory medical school curriculum.

**Conclusions:** A course of Meditation and Massage may be a valuable complementary elective to medical school education, supporting self-care and stress management in preparation for a demanding profession, and may improve palpatory examination skills.

## Introduction

Two key goals compelled us to implement an innovative curriculum called Massage & Meditation. First, it had been suggested that there is a need for educating medical students in wellness self-care and skills for life-work balance ( Dyrbye, Thomas and Shanafelt, 2005) in order to prevent student and physician burnout, which medical students are expected to encounter as a major challenge during residency and later clinical work ( Hochberg *et al.*, 2013). Secondly, the current clinical training in medical schools has been criticized for emphasizing and relying too heavily on the use of imaging and molecular technologies for clinical assessment and diagnosis, leading to significantly diminished time spent developing hands-on palpatory skills, which are therefore declining among medical graduates ( Verghese and Horwitz, 2009). A thorough physical exam includes skillful touch. Yet with increasing time constraints in clinical settings, patients often express that clinicians spend more time attending to their computers than actually touching or examining the physical sites of pain. It has been suggested that this may take away from a trusting clinician-patient relationship ( Zaman, Verghese and Elder, 2016), and may even be a risk factor for medical errors ( Verghese *et al.*, 2015). Is the next generation of computer-savvy millennial physicians at risk of being “out of touch”?

A systematic review of benefits from massage has shown that massage produces its largest effect size for reducing anxiety ( Moyer, Rounds and Hannum, 2004; Field, 2014), possibly through mechanisms affecting the physiological stress response and oxytocin ( Morhenn, Beavin and Zak, 2012; Tsuji *et al.*, 2015), and supporting embodiment ( Farb *et al.*, 2015). In medical education settings, meditation and mindfulness have demonstrated beneficial preliminary results for self-care and burnout prevention ( Warnecke *et al.*, 2011; Shakir *et al.*, 2017; Daya and Hearn, 2018). We aimed at exploring whether a course combining massage and meditation could give medical students tools for self-care and skillful touch that would be applicable in their professional and personal lives.

In 2005 the Dean’s Office of our Medical School approved the Massage & Meditation curriculum as an elective course with credit. The course was developed by one of the authors (WM) and has since been offered one to two times every academic year. For the past 13 years, approximately 10% of each class of medical students has completed the course. We decided to generate a comprehensive description of the curriculum’s components and analyze the quantitative and qualitative course evaluations in order to determine if medical students were perceiving benefits from the course for their emotional well-being and/or for their confidence in touching patients. In addition, we wanted to explore whether interoceptive bodily awareness, the perceived sense of the physiological condition of the body ( Craig, 2003) and a potential mechanism for improving self-care( Farb *et al.*, 2015), could appreciably change over a course of Massage & Meditation. For this latter aim, for two consecutive courses, we conducted a survey with course participants that was approved by the university’s Institutional Review Board.

## Methods

### Development and Description of the Course Curriculum

All classes were led by a MD-physician on faculty at the University’s Medical School (WM). He developed his expertise though several weeklong massage courses and had completed training in Manual Medicine in Germany, a board-certified post-graduate subspecialty training for physicians comparable to the hands-on training in US osteopathic colleges. Additionally, he learned Esalen ( Mehling and Mehling, 1990)-style massage techniques in an extended work scholar program at Esalen Institute, Big Sur, CA, and taught with the Esalen Massage school faculty. The curriculum was structured similar to an abbreviated version of a weeklong workshop for Esalen Massage ( Mehling and Mehling, 1990) with more details in functional anatomy for medical students.

The objectives of the course were to train students to provide a 1-hour full-body massage to peers, and to teach them basic skills in meditation. Combining both massage and meditation was intended to add an active educational element to a generally more passive, receptive massage experience. Both giving and receiving a massage may create a short-term sense of embodied physical well-being, the sensory awareness of which may be deepened and enhanced by a body-focused meditation practice. The course was announced as an elective for student self-care, for facilitating musculoskeletal palpatory skills and experiencing the benefits of massage first-hand on their own bodies.

The weekly evening classes lasted 2 hours over initially ten and later nine weeks. Classes were experiential and hands-on. Students were given handouts with literature from medical journals regarding history, theory, and research studies of massage upon request ( Cassileth and Vickers, 2004; Moyer, Rounds and Hannum, 2004; Mehling *et al.*, 2007; Furlan *et al.*, 2009; Crane *et al.*, 2012; Field, 2014). The first class (class #1) introduces logistics, dressing recommendations, norm-setting, and explicit and strict safety rules: as students are partially unclothed to receive a massage, they were asked to consent-by raising hands-to 1) confidentiality regarding other students’ physical body characteristics, 2) abstaining from exhibitionism and erotic stimulation, 3) respecting everybody’s personal level of ease or unease with being seen or touched, and 4) following a specific method for holding up a large sheet to protect their partner during dressing and undressing. This consent was required to provide a safe class environment and respect modesty. Students were encouraged to switch partners from class to class to allow the experience of a variety of bodies and touches. The first class ends with a “sandbagging” exercise (adapted from Trager® massage ( Ramsey, 1997)), in which one student moves the body parts of another student as if they were bags of sand to be shaken out, learning to hold the other in a way that she/he notices where tension is held, is able to trust, let go, and relax. After 20 minutes, they switch roles so that everybody experiences both sides of the exercise, giving and receiving.

The subsequent five classes (classes #2-6) follow an identical basic structure. Classes begin with a 15-minute guided body scan and breath awareness sitting meditation, and end with a 5-minute meditation intended to draw attention to what may have changed in their mental and physical state from before to after the massage. Thus, the experience of giving and receiving a massage was framed by a mindful awareness practice in order to augment the usual rather passive massage experience with an educational element: a training to perceive subtle changes in interoceptive sensations, the core of mindful bodily awareness ( Mehling *et al.*, 2009). Following the initial meditation practice, the instructor (WM) demonstrated massage techniques on a student for a particular section of the body (e.g., back or head/neck), including details on anatomical landmarks, functional anatomy, and biomechanics. The massage techniques were meant to support relaxation, wellbeing, sensory awareness, and a sense of flow, wholeness, and integration, alternating between more specific moves and long, slow integrating strokes adapted from the Esalen style ( Mehling and Mehling, 1990). While the students practiced the massage moves, the instructor rotated from table to table to assist and correct as needed. The light in the room was dimmed, and calming, “neutral” instrumental music was played, chosen to minimize possibly loaded associations with specific traditions or memories (e.g., Arvo Pärt, Anouar Brahem). The final 5-minute meditation primarily focused on sensory awareness of the body and breath.

For the two classes (classes #7-8) following these five sessions, the students were able to give a 70-minute full-body massage, again framed by the meditation practice, for which verbal guidance was gradually reduced to a minimum. The last class (class #9) of the course was dedicated to clinical applications, specifically to the manual treatment of musculoskeletal pain and trigger points in a gentle compression style ( Shah and Gilliams, 2008).

Over the years the course has been offered, students requested only minor changes to the rules and the general structure of the class. In response to student feedback, the six-session content on *partial* massage of parts of the body was condensed, reducing the total number of sessions from ten to nine. Few movements, such as range of movement for the hip joint in supine position, were eliminated due to lack of practicality and student discomfort. A few movements from Trager® and Rolfing®( Jones, 2004) were added later as well as the more detailed focus on muscular trigger point in the final class. Again responding to student feedback, in each class students received a 1-page handout that outlined the massage strokes practiced in class. Following another student request, the course director recently produced a 50-minute video of the complete massage available on the UCSF Institute website that includes a choice of music audio https://youtu.be/rC_X4BY1nII or verbal instructions for the massage strokes https://youtu.be/l0sCZAH2ZwI.

The Medical Student Participants and their Recruitment

The course was regularly announced in the medical student curriculum sponsored by the Department of Family and Community Medicine. Two students, who had taken the class before, volunteered to serve as student organizers and sent a brief announcement to their peers in the first and second year. They accepted 12 participants. Third- and fourth-year students’ clinical rotations precluded commitment to a weekly class. In order to support trust and confidence-building among peers in a group setting, classes were limited to medical students only. The class was pass/fail; students were allowed to maximally miss one class. Class participants were representative of the student body at our Medical School: 52% female; 33% underrepresented; 60% Asian; 42% White; 36% Hispanic; 24% African-American; 4 Hawaiian/Pacific Islander; 1% American Indian/Alaskan; 9% Not Specified ( Education and Medicine, 2018).

### Sources for quantitative and qualitative course evaluation

• Since 2005, at the conclusion of every course, students filled out an anonymous 5-item standard course evaluation. Items were statements adapted from other common student course evaluations (e.g., “The speaker effectively presented the subject matter”) on a 5-point Likert scale. The bottom of the questionnaire left ample space for notes: “Comments (E.g., How could this course be improved? What inspired you most?)”
• For the two classes in Fall 2017 and Winter 2018, students signed informed IRB-approved consent to partake in a 11-item pre and 32-item post survey with questions about their knowledge of and confidence in practicing massage and meditation, and on the value of the learned for their private and professional life (using a 6-point Likert scale). Examples are “Are you able to describe some of the basics about meditation?” or “How confident do you feel about touching an undressed patient?”
• In order to determine whether the course changed interoceptive bodily awareness, the Multidimensional Assessment of Interoceptive Awareness (MAIA)( Mehling *et al.*, 2012) was included in the surveys. The MAIA is a self-report instrument designed to capture potential changes in interoceptive bodily awareness with mind-body approaches, has shown sensitivity to change with body and breath-focused meditation ( Bornemann *et al.*, 2014) but has not yet been applied with massage. It includes 32 items on eight scales (on a 6-point Likert scale) for Noticing; Not-Distracting; Not-Worrying; Attention Regulation; Emotion Regulation; Self-Regulation; Body Listening; and Trusting.

### Analysis Methods

Quantitative: all paper and pencil surveys were anonymous and did not include ID-numbers. We are only able to report descriptive data with mean values for the 5-item course evaluations over 13 years and for the pre-post surveys for two courses between September 2017 and March 2018. Because we did not include IDs in these surveys and thus were unable to pair pre and post responses, we report group means without *p*-values abstaining from further statistical analyses.

Qualitative: We employed applied thematic analysis of the free written responses in the surveys. Applied thematic analysis is an inductive and analytical methodology used to develop a theory of the phenomenon being studied allowing core problems and processes to emerge from the analysis, as opposed to applying external concepts to the data ( Guest, MacQueen and Namey, 2012). The findings were modeled in an iterative process that allowed for continual modification to best explain the meaning of the data.

All surveys were independently coded by MB, LM, and WM to develop a codebook that was then condensed and elaborated upon. Surveys were recoded with the final codebook by MB and LM independently to create an interrater reliability check. LM first coded one third of the quotes. MB and LM discussed any code differences until reaching 100% agreement. LM subsequently coded the remaining two thirds of quotes independently. For some of the quotes that could be coded in multiple ways, only identical codes were counted as agreements (numerator) with the total number of all given codes as denominator. The final interrater reliability was 81%. Data interpretation and analysis were written as memos and linked to specific codes. The relationships among these categories were explored through iterative conceptual modeling and tested against the survey data for its completeness and explanatory power. Codes were repeatedly modified until we agreed on a final set of themes, that best captured and represented the meaning of the students’ feedback.

## Results/Analysis

### Quantitative Results

In total, we collected 181 anonymous course evaluations. These were generally given at the end of the last class that was not always attended by all of the 220 students due to end-of-semester test preparations. Using the university’s standard evaluation tool, the students gave the course an overall evaluation score of 4.94 [Range 1-5] ( Table 1). Asked whether the course should be offered next year, all 181 answered “absolutely” (= 5).

**Table 1.** Survey results pre and post classes.

	**pre**	SD	**post**	SD	Δ
Are you able to apply massage?	**2.38**	1.50	**4.29**	0.55	+1.91
Are you able to apply meditation?	**2.83**	1.17	**3.92**	0.78	+1.09
How confident do you feel about touching a dressed patient?	**3.88**	1.03	**4.71**	0.55	+0.83
How confident do you feel about touching an undressed patient?	**2.63**	1.21	**4.25**	0.74	+1.62
How confident do you feel about being able to discern different levels of tension in another person’s muscles?	**1.83**	1.43	**3.46**	0.93	+1.63
How confident do you feel about being able to apply a basic meditation in your life?	**3.00**	1.25	**3.96**	0.75	+0.96
Does the concept of embodiment have meaning to you?	**2.13**	1.78	**3.63**	0.92	+1.50
Does the concept of body awareness have meaning to you?	**3.50**	1.50	**4.50**	0.59	+1.00
Do you see any value in distinguishing between thinking about versus sensing your own body?	**4.00**	1.38	**4.54**	0.59	+0.54
Are you able to describe some of the basics about meditation?	**2.00**	1.41	**4.75**	0.53	+2.75
Are you able to describe some of the basics about massage?	**3.29**	1.43	**4.39**	0.72	+0.90
How much did you enjoy the training?			**4.92**	0.28	
Do you consider the training relevant?			**4.79**	0.51	
Was it a good use of your time?			**4.88**	0.34	
Will you apply what you learned?			**4.71**	0.62	
Would you recommend the classes to your peers?			**4.96**	0.20	
Are you content with the amount you learned?			**4.54**	0.59	
Did what you learned meet what you expected to learn?			**4.54**	0.51	
Will you apply what your learned in class?			**4.58**	0.78	
Did you already apply skills you learned in class?			**4.17**	1.24	
Are the skills relevant for your personal life?			**4.67**	0.48	
Are the skills relevant for your professional life?			**4.38**	0.71	
Are you aware of any change in your personal activities related to what you learned in class?			**3.92**	0.78	
Are you aware of any change in your professional performance related to what you learned in class?			**3.67**	1.05	
Are you able to demonstrate some of the massage movements?			**4.83**	0.38	
Did you learn anything new in practical anatomy that is of clinical value for you?			**4.04**	1.08	
Are you able to describe some of the basics about meditation?			**4.42**	0.78	
Did the speaker effectively present the subject matter?			**4.83**	0.38	
Did the course stimulate your interest in this topic?			**4.92**	0.28	
Did you learn something new?			**4.96**	0.20	
Should this course be offered again next year?			**5.00**	0.00	
Was the course overall of value for you?			**4.92**	0.28	

**Scale range** 0 (for “not at all”) to 5 (for “completely/ absolutely”)

The scores of all 24 students of the classes that completed the longer survey and the MAIA are shown in Table 1. Average rating was >4.9 [Range 0-5] for the questions “Would you recommend the classes to peers?”; “How much did you enjoy the training?”; “Was the course overall of value for you?”; and “Did the course stimulate your interest in the topic?” The students considered the training highly relevant (4.79), felt the course was a good use of their time (4.88), were confident that they are able to demonstrate some of the newly learned massage movement (4.83) and plan to apply what they learned (4.71). All items asked both before and after the course increased in their scores (all ranges 0-5). The highest increases were found for the self-reported ability to describe some of the basics of meditation (+2.75) and for the ability to apply a massage (+1.91). These were followed by two score changes that are of clinical relevance, the confidence in touching an undressed patient (+1.62) and their ability to discern different levels of tension in another person’s muscles (+1.63). Students reported at baseline that they were relatively new to the basics about meditation (2.00) and the concept of embodiment (2.13), for which mean scores improved to 4.75 and 3.63, respectively. Students felt that the skills were highly relevant for their personal (4.67) and professional life (4.38).

Mean scores on seven of the eight MAIA scales ( Table 2) improved over the course. Before classes, students gave scores of >3 [Range 0 to 5] for Noticing, Emotion Regulation, and Trust and relatively lower scores for Attention Regulation (2.54) and Body Listening (2.42). The latter two and Self-Regulation improved on average by more than 1 point. For Not-Distracting and Not-Worrying scores did not change appreciably.

**Table 2.** MAIA results: Values are on a scale from 0 (never) to 5 (always) and indicate how often statements apply generally in daily life.

**MAIA Scale**	**pre**	**post**	pre-post change of means	#pre/post
	mean (SD)	mean (SD)		
Noticing	3.29 (0.65)	4.02 (0.55)	+0.73	24/24
Not-Distracting	2.98 (0.91)	2.79 (0.97)	-0.19	22/24
Not-Worrying	2.64 (0.82)	2.83 (0.62)	+0.18	24/24
Attention Regulation	2.5 (0.90)	3.63 (0.61)	+1.09	24/24
Emotion Regulation	3.47 (0.81)	4.24 (0.49)	+0.77	24/24
Self-Regulation	2.90 (0.93)	4.00 (0.51)	+1.10	24/24
Body Listening	2.42 (0.98)	3.49 (0.83)	+1.07	24/24
Trusting	3.24 (0.93)	4.19 (0.74)	+0.95	24/24

SD: Standard Deviation; #: number of complete observations.

### Qualitative Results

In total, 174 of 181 students gave written responses in the comments section of the survey. The themes that emerged from the data fell into six categories as described below.

**Self-care and stress management:** The most prominent theme was that meditation and message can serve as tools for self-care and stress management. Both meditation and massage heighten students’ awareness. Students captured this sentiment in explaining how this course gave them a new understanding of themselves and made them more self-reflective:

“ *The class focus on the whole body, awareness, and presence both as a practitioner and as a person being worked with was incredible. I feel a whole world was opened up to me in understanding my body and the whole body.*” (#130)

*“[It] has given me a new tool to manage anxiety and re-evaluate my relationship with my body and my consciousness.*” (#109)

Students commented on their experiences with both giving a massage and receiving one. Many students were surprised and thought that it was a paradox how after doing actual work (giving a message) they felt more energized rather than fatigued, an experience quite different from usual clinical work:

“ *I was so surprised and pleased with how good it felt to GIVE massage. I had expected receiving to me the highlight but I found that I enjoyed giving more.*” (#24)

Many students made statements about more clearly differentiating between being in their body versus in their mind:

“ *I particularly enjoyed getting massage - it was the time [of] my day that [I] was really in my body and not head.*” (#16)

Massage pulled them from their thoughts and grounded them in the sensations they felt in their hands or body, in a way, heightening their experience of feeling connected to their body:

“ *The best part of the class..was just leaving my thoughts and worries behind and focusing on the movements of my hands.*” (#58)

Students emphasized the

“ *importance of being present in my own body for my own health and being.*” (#30)

They reflected on the comfort of receiving a massage,

“ *It made me appreciate the power of communication through touch and how reassuring it can be to have someone simply place their hands on you.*” (#160)

Students focused on touch and meditation:

“ *The power of touch reminded me how healing touch can be, and how renewed and energized it made me [to] connect with others in this way.”* (#40)

“ *Learning some concrete meditation techniques has been very helpful for stress management and for falling asleep.*” (#89)

“ *The meditation practice has given me a new tool to manage anxiety and re-evaluate my relationship with my body and my consciousness.” (*#109)

**Comfort with touch:** One student added a single sentence in her evaluation’s Comment section:

“ *I feel much more comfortable touching people.*” (#56)

Learning about touch through massage stood out, as many students felt that it helped make them more comfortable in their skin and bodies as well as more comfortable touching others, notably for application in the clinical setting:

“ *I find it extremely difficult to be comfortable in my own body..and I think that translates to how I interact with others’ bodies as well. I found this experience to be so therapeutic, so loving, and so very healing.*” (#167)

Multiple students expressed how initially they had poor self-images of their bodies and had to overcome some resistance against being exposed and touched. Student became increasingly comfortable by undressing and allowing classmates to massage them,

*“[I] had to face..each session and overcome any preconceptions I had about my body.*” (#159)

“ *The most valuable part was getting comfortable touching other people. That basic thing - touch - is almost absent from our curriculum and it is why I took the class. It was definitely hard and anxiety-provoking at first, but I got better at it.”* (#77)

Students expressed that the course experience encouraged them to use more touch with patients.

**Clinical applicability:** At least a quarter of the students commented on the practicality of the massage and meditation techniques:

“ *I think the guided meditation in the beginning of the sessions went a long way towards training us how to be mindful of our surroundings and granted us a skill-set we can not only use for ourselves, but [for] future patients and peers as well.*” (#32)

“ *WM ..* *inspired me to use touch in my future medical practice*”. (#70)

*“* *I have a new appreciation for the use of massage in medicine. I come from a family of physicians, but they’ve largely taught me about curing diseases with pills/medicines. It’s important for me to learn about the other medical techniques (such as massage - trigger points!) that can be used to help my patients.”* (#162)

Some students desired to actively practice massage,

*“I’m inspired to use my hands as new tools in the healing process. I love the warmth I feel now [in] my ever-cold hands after I give a massage.”* (#29)

**Comparison to other parts of medical school:** Students drew comparisons between general medical school teaching and this course and expressed that learning self-care methods and the “power of touch” are elements missing in medical school education. Many expressed how massage is a great way to review anatomy. Others explained that the course contributed to their medical education in ways the standard medical curriculum did not.

“Relaxation and feeling one’s body deeply are important for maintaining and promoting one’s health - this is something that is not discussed in our standard curriculum.” (#161)

Some students expressed how this course taught them truly applicable skills:

*“I was really amazed with my ability to perform an hour-long massage on a classmate by the end of the course..This is one of the few times where I felt like I learned something ‘useful’ in medical school.”* (#83)

**Community building:** Students explained how their group felt like a community:

*“* *I love how this class is always a safe space for us to give and receive acts of service (massages) that can feel very intimate. It is a gift to be able to connect with other people in this way.”* (#126)

“ *I have been incredibly inspired by the positive energy of all the participants and .. how healing touch can be. We spend a lot or very little time with one another on campus depending on whether we are in classes together, but coming here as a smaller group and in a safe and intimate setting. It has been incredible to get to know people in a different way.*” (#45)

The community was created through students making themselves vulnerable, trusting each other, and having unique experiences different from their peers’ who were not in the course.

**Learning modalities:** Students appreciated the general format of this course. They noted how listening and observing, and then immediately applying that knowledge in an experiential, hands-on way was helpful. Switching between giving a massage and then receiving it, or vice-versa, was a compelling way to acquire the skills:

“ *So awesome to have an experiential, hands-on class. Listening and then immediately trying is such a great way to learn. I am getting a massage table and really want to keep it up and learn more.*” (#18)

“ *I think the guided meditation in the beginning of the sessions went a long way towards training us how to be mindful of our surroundings and granted us a skill-set we can not only use for ourselves, but future patients and peers as well. The course is structured well as is (is there really any other way to teach that doesn’t involve moving from [one] region of the body to another?)*” (#81)

Some students suggested improvements to the course such as adding massage for a seated person, as larger massage tables are generally not available in clinic spaces. One student suggested:

I really liked the stepwise progression of massage techniques and the integration of teaching and learning in one session. I would like the trigger point session earlier in the course, because it is so relevant as a daily skill." (#111)

## Discussion

To our knowledge, this is the first report about a medical school curriculum that combines massage and meditation. This is a summary of the student responses to this curriculum over a 13-year period. The responses are based on regular student evaluations with free space for comments as well as a pre-post survey we conducted for two consecutive classes.

In summary, the students that elected this course appeared highly satisfied with the class. They self-report having gained confidence in being able to apply massage and meditation in their personal and, to a limited degree, in their professional life. They commented on the importance of touch, and that they gained more confidence in touching each other as well as undressed patients. They seemed to have understood the value of interoceptive bodily awareness, mindfulness and embodiment and expressed a desire to apply their skills in future clinical work. They learned that shifting one’s attention from mental activity to sensing the body can help in reducing stress. They felt that they developed new skills for self-care and stress management, experienced a sense of community among peers, and stated that the class provided necessary teaching as a complement to the mandatory medical school curriculum.

To our knowledge, there exists no similar medical school curriculum for massage in the US. We can only compare the self-reported benefits of the meditation component of this class to other school curricula on mindfulness or meditation. Students exposed to 8 to 10 weeks of a Mindfulness-Based Stress Reduction (MBSR)( Irving, Dobkin and Park, 2009) class or other extensive mindfulness-based programs ( Hassed *et al.*, 2009) experience benefits for physical and psychological health and well-being( Dobkin and Hutchinson, 2013). Despite its brevity, the reported improvements in interoceptive bodily awareness, as assessed by the MAIA, are in line with reports for meditation studies ( Bornemann *et al.*, 2014; Fissler *et al.*, 2016). In addition to the increase in self-reported interoceptive bodily awareness, the value of learning a mindful style of bodily awareness for relaxation, emotion regulation (anxiety) and stress management was supported by the students’ qualitative statements. This aspect of the course teaching is a core element of any meditation or mindfulness training ( Holzel *et al.*, 2011; Farb *et al.*, 2015). The MAIA showed the largest improvement in Self-Regulation, the ability to regulate distress by attention to body sensations; Body Listening, active listening to the body for insight; and Attention Regulation, the ability to sustain and control attention to body sensation. Scores for Not-Worrying, the tendency not to worry or experience emotional distress with sensations of pain or discomfort, did not improve strongly, which may be due to the younger age and general health of our participants. Alternatively, this may be due to the relatively low consistency reliability found in several studies with application the MAIA for these two scales, which prompted a revised MAIA-2 version ( Mehling *et al.*, 2018).

We do not know whether learning skillful touch and developing confidence in touching others will translate into changes in the future physicians’ attitudes and clinical skills and, in turn, potentially improve medical care quality. Costanzo and Verghese state: “The privilege of examining a patient is a skill of value beyond its diagnostic utility. A thorough physical examination is an important ritual that benefits patients and physicians. The concept of embodiment helps one understand how illness and pain further define and shape the lived experiences of individuals in the context of their race, gender, sexuality, and socioeconomic status. Understanding ritual in medicine, including the placebo effects of such rituals, reaffirms the centrality of the physical examination to the process of building strong physician-patient relationships” ( Costanzo and Verghese, 2018). Touch may be a central element of ritual in physician-patient communication and worth including in medical school curricula where it may be of equal benefit for both patients and physicians.

We are aware of the imitations of this study. Due to our omission of using study IDs for our anonymous surveys, we were unable to apply appropriate statistical tests for paired data. We can only present mean values for all pre and post observations of the classes. We did not receive student evaluations from all students of the past years, as numerous students missed the last classes due to exams, travel, or other commitments. However, for the survey we conducted during the last two courses we received surveys from all 24 participants. Standard deviations for the higher post-class survey scores were smaller than for pre-class scores. These are likely due to ceiling effects and do not permit further inferences. Generalization of course benefits may be limited due to the massage being taught by a MD faculty instructor, who was highly trained in manual medicine skills including soft tissue massage. However, we believe that an osteopathic physician, a physician that has prior training as a massage therapist, or a physician who has taken courses in massage techniques and is joint by a massage practitioner for teaching similar classes, can teach such a course, provided the Medical School has massage tables available. The MAIA is a self-report measure of interoceptive bodily awareness and scores may potentially be sensitive to cognitive learning during the class rather than interoceptive awareness itself. Qualitative results of a one-time questionnaire are limited by not having asked follow up questions to better understand the students’ responses, statements and opinions.

## Conclusion

A course of Meditation and Massage, as taught for over 10 years at our university, may be a valuable complementary elective to medical school education. It may support self-care and wellbeing in students, support stress management in preparation for a demanding profession, and may improve examination skills and physician-patient relationships. Further research is necessary to evaluate the potential benefits of the Massage & Meditation curriculum.

## Take Home Messages

In response to the need for supporting medical students’ well-being and self care, we developed a curriculum for first and second year medical students: Massage & Meditation. This study evaluates the curriculum and the students’ commentaries, using quantitative and qualitative data. The findings suggest that medical students may benefit for their personal and professional life from such a curriculum.

## Notes On Contributors

**Maxwell Binstock** is a 4 ^th^ year medical student with a research background in qualitative methods and a passion for integrative medicine. This study was part of a student research project supervised by Dr. Mehling.

**Leora Morinis** is a 2 ^nd^ year medical student, who took part as a student organizer in the massage class and is interested in medical research. This study was part of a student research project supervised by Dr. Mehling.

**Shelley Adler**, PhD, is Professor in the Department of Family and Community Medicine and the director of the UCSF Osher Center for Integrative Medicine. She trained in medical anthropology, sociocultural gerontology, and medical education research. In addition to her role as director of the Osher Center, she is an integrative medicine researcher and educator, examining inequalities and exploring avenues for equity in health and education. Dr. Adler is also involved in teaching, curriculum development, advising and mentoring, and educational administration and leadership. She co-directs the Osher Center’s research fellowship program and teaches medical student and interprofessional courses in integrative medicine and integrative end-of-life care. Dr. Adler was inducted into the UCSF Haile T. Debas Academy of Medical Educators in 2005 and served as the Osher Center’s director of education from 2008 to 2019.

**Wolf E. Mehling**, MD, is Professor in the Department of Family and Community Medicine and faculty at the UCSF Osher Center for Integrative Medicine. Specialized in non-pharmacological approaches to chronic pain, he provides medical services at the UCSF Medical Center and teaches medical students and residents. His research focuses on mind-body therapies including integrative exercise for chronic back pain, PTSD and Alzheimer’s disease. He is particularly interested in interoceptive bodily awareness.
