# Supplementary material for: Students’ response to a Massage & Meditation medical school course elective
Source: MedEdPublish (2016). 2020 Feb 13;9:28. [Version 1] doi: 10.15694/mep.2020.000028.1 (PMC10697436; doi:10.15694/mep.2020.000028.1)
Supplement: Peer review history

# Reviewer report: Reviewer response for version 1

**DOI:** 10.21956/mep.19966.r30346

**Published:** 2020-04-13

**Authors:** P Ravi Shankar

**Affiliations:** 1 American International Medical University

This review has been migrated. The reviewer awarded 5 stars out of 5 This is an interesting and very well-written and presented study. The authors describe a massage and meditation elective for medical students. The consent procedure described is interesting and helped to create a safe class environment. Was any written consent obtained? I am happy to note that many of the authors are medical students who had participated in the program. The authors can describe interoceptive bodily awareness in greater detail. Were there any special instructions or guidelines to be followed while providing massage to a student of the opposite gender? A more detailed description of the eight scales on the MAIA will be useful to readers. I may also be interested in more details of the meditation component of the course. The authors have described the methodology, the study findings and the limitations of the study well. The free-text comments are particularly illuminating. This article will be of broad interest.

Reviewer Expertise:

NA

No decision status is available

# Reviewer report: Reviewer response for version 1

**DOI:** 10.21956/mep.19966.r30344

**Published:** 2020-02-24

**Authors:** sathyanarayanan varadarajan

**Affiliations:** 1 SRM Medical College, SRM University

This review has been migrated. The reviewer awarded 5 stars out of 5 In this enchanting article the authors, for the first time, explore whether a course combining massage and meditation could give medical students tools for self-care and skillful touch that would be applicable in their professional and personal lives.They developed and implemented a curriculum for first and second year medical students (Massage & Meditation) as an elective and evaluated the curriculum and the students' commentaries using quantitative and qualitative data, which consist of the student responses over a 13-year period..!In summary, the students that elected this course appeared highly satisfied with the class. They self-report having gained confidence in being able to apply massage and meditation in their personal and, to a limited degree, in their professional life.The authors conclude that this course may be a valuable complementary elective to medical school education. It may support self-care and well-being in students, support stress management and may improve examination skills and physician-patient relationships. I heartily congratulate the authors as this is a very valuable study done in an extensive way. Though there are certain limitations, it has opened new doors towards further research in this field. Its contribution is highly significant as there is not much scientific evidence available in the area of Meditation in Medical education... This study will be helpful to all the medical educators who are involved in curriculum design & development …

Reviewer Expertise:

NA

No decision status is available

# Reviewer report: Reviewer response for version 1

**DOI:** 10.21956/mep.19966.r30345

**Published:** 2020-02-16

**Authors:** Virginia Randall

**Affiliations:** 1 Uniformed Services University of the Health Sciences

This review has been migrated. The reviewer awarded 5 stars out of 5 I enjoyed reading this well written article and picturing the course taking place in our medical school.As for touching patients, as a pediatrician that was the highlight of the exam and often the only way to communicate with the patient. I think the physician who led the course was incredibly well trained and charismatic as well. so I wonder whether these results could be achieved elsewhere? Regardless, the course should be offered in every medical school following the privacy principles given here and with qualified instructors.

Reviewer Expertise:

NA

No decision status is available

# Reviewer report: Reviewer response for version 1

**DOI:** 10.21956/mep.19966.r30347

**Published:** 2020-02-14

**Authors:** Felix Silwimba

**Affiliations:** 1 University of Lusaka

This review has been migrated. The reviewer awarded 5 stars out of 5 this study is informative and is worth considering in conducting medical education in low middle income countries . it will need modification to suit the sociocultural requirements.

Reviewer Expertise:

NA

No decision status is available
